# SARS-CoV-2 likely targets cellular PDZ proteins: a common tactic of pathogenic viruses

**DOI:** 10.2217/fvl-2020-0365

**Published:** 2021-05-19

**Authors:** Andrew P Rice, Jason T Kimata

**Affiliations:** ^1^Department of Molecular Virology & Microbiology, Baylor College of Medicine, Houston, TX 77096, USA

**Keywords:** coronaviruses PDZ proteins, E protein, Orf3a protein, PBM, PDZ binding motif, SARS-CoV-2, viral pathogenesis

At the time of this writing, SARS-CoV-2 has infected over 110 million individuals and over 2 million deaths have occurred. While there have been significant advances in understanding the pathogenesis of COVID-19, considerably less is known about how SARS-CoV-2 proteins interact with host cell proteins to facilitate viral replication, dissemination and transmission. One way in which pathogenic viruses enhance replication is through targeting and perturbing function of cellular PDZ proteins. This commentary focuses on two SARS-CoV-2 proteins – E and Orf3a – that contain a protein binding domain at their carboxyl termini that is predicted to target a family of cellular PDZ proteins. Based on findings from many viruses, including SARS-CoV-1, the targeting of cellular PDZ proteins appears to be a common mechanism critical for viral pathogenesis [1,2]. It is likely that the abilities of the E and Orf3a proteins to target PDZ proteins also make important contributions to the pathogenesis of SARS-CoV-2. The PDZ domain is a protein–protein interaction module that is wide spread in the human proteome – over 100 human proteins contain one or more PDZ domains [1]. PDZ domains are bound directly by specific peptide sequences located at the C-terminus of a target protein. The peptide sequence is called a PDZ-domain binding motif (PBM). PBMs are grouped into three specificity classes: type I PBM (-X-S/T-X-ϕ_COOH_), type II PBM (-X-ϕ-X-ϕ_COOH_) and type III PBM (-X-D/E-X-ϕ_COOH_), with ϕ representing a hydrophobic residue. Cellular PDZ domain proteins are involved in several processes of significance to viral infection: maintenance of cell-cell junctions, cellular polarity and signaling pathways [1,3–5]. Viruses commonly encode proteins with a PBM that can bind directly to multiple cellular PDZ proteins to increase viral replication and spread. For example, the influenza A virus NS1 protein contains a PBM that targets the Scribble protein; thereby, protecting infected cells from apoptosis [6]. The NS1 PBM also targets Dlg1 to disrupt cellular tight junctions, likely increasing viral spread within and between individuals [7,8]. Because the PBM consists of only four amino acid residues at the C-terminus of a protein, it seems that selection pressures allow viruses to rather easily append a PBM to the C-terminus of a viral protein if this can confer an evolutionary advantage.

The E protein of SARS-CoV-2 and SARS-CoV-1 are 98% identical and contain a type II PBM – DLLV – at their carboxyl termini. The SARS-CoV-2 and SARS-CoV-1 Orf3a proteins are 72% identical and contain a type II PBM – SVPL – at their carboxyl termini. In contrast, the MERS E protein contains a type III PBM at its C-terminus with the sequence DEWV. MERS encodes only a single Orf3 protein, unlike SARS-CoV-1 and SARS-CoV-2 which encode Orf3a and Orf3b proteins. The MERS Orf3 protein does not contain a PBM.

The PBM of the E protein from SARS-CoV-1 contributes to viral pathogenesis in a mouse model. Deletion or mutation of the PBM of this E protein has a minimal effect on viral replication in Vero cells but reduces replication in Huh-7 and CaCo-2 cells by 200-fold [9]. Mutation of the PBM in the E protein has a dramatic effect on infections in mice. Infections with SARS-CoV-1 containing the wild-type E protein PBM results in severe weight loss and 100% mortality of mice. In contrast, infections with a virus containing a mutated E protein PBM results in moderate or no weight loss and 100% survival of mice [2]. Thus, the PBM of the E protein determines whether mice live or die from SARS-CoV-1 infection. The lungs of mice infected with the wild-type virus are highly edematous with substantial leukocyte infiltration. Mice infected with a virus encoding a mutated E protein PBM have only minimal edema and leukocyte infiltration.

Deletion of the SARS-CoV-1 Orf3a protein PBM has no effect on replication in Vero cells and little effect on weight loss or mortality in mice [10]. The contribution of the SARS-CoV-1 Orf3a protein to viral pathogenesis is therefore unclear. However, an analysis of over 70,000 high-quality SARS-CoV-2 genomes did not observe any mutations in the PBM of Orf3a, suggesting a critical function of the Orf3a PBM *in vivo* [11]. It is notable that the Orf3a protein PBM is required for viral replication in Vero cells in a virus with a deletion of the E gene. Thus, the Orf3a protein PBM possesses an important viral function *in vitro* that compensates for a deletion of the E protein.

SARS-CoV-1 viruses were constructed in which Orf3a was deleted and the E protein either contained a termination codon preceding the PBM, or the entire PBM was deleted. Infections in Vero cells resulted in rapid reversions of both viruses – the mutant virus lost the premature termination, while the virus with a deleted PBM acquired an alternative PBM at the E protein C-terminus with the sequence ALAV (type II PBM). These genetic experiments demonstrate the critical role of the E protein PBM in viral replication and show that key functions of viral PBMs can be investigated *in vitro* [10].

To investigate the role of the PBM in the E and Orf3a proteins of SARS-CoV-2, it will be necessary to identify the PDZ proteins that serve as targets for this binding domain. A relatively straightforward approach is to express the two proteins in a biologically relevant lung epithelial cell line – perhaps Calu-3 or A549 – and utilize immunoprecipitation and mass spectrometry to identify cellular proteins that co-immunoprecipitate with the viral proteins. To identify cellular proteins that require the viral PBM for immunoprecipitation, E and Orf3a with mutations in the PBM will be essential controls. With this strategy, proteins that co-immunoprecipitate with the wild-type but not PBM mutant proteins will identify cellular proteins whose association with the viral proteins requires a PBM. These cellular proteins are highly likely to contain PDZ domains, although non-PDZ proteins will be found in co-immunoprecipitations if they exist in a multiprotein complex with a PDZ protein. The analysis of recombinant viral and cellular PDZ proteins *in vitro* can demonstrate direct protein–protein interactions, as was demonstrated for the influenza virus NS1 protein and Scribble and Dlg1 [6,7]. A recent publication by Krogan and colleagues used immunoprecipitation/mass spectrometry technology to identify the set of cellular proteins that target the SARS-CoV-2 proteins [12]. Unfortunately, these investigators added a Strep tag at the C-terminus of both the E and Orf3a proteins, which masks the PBMs, preventing identification of associating cellular PDZ proteins.

It should be noted that the SARS-CoV-1 E and Orf3a proteins are also viroporins – viral proteins with ion channel activity. The viroporin function of the SARS-CoV-1 E protein is required for maximum viral replication and virulence in mice, whereas the SARS-CoV-1 Orf3a viroporin activity is required for maximum replication in mice but does not make a major contribution to pathogenesis [10]. Given the conservation of these two proteins between SARS-CoV-1 and SARS-CoV-2, it is likely that the E and Orf3a proteins from SARS-CoV-2 are also viroporins and in the case of the E protein, this function is likely to be important for viral pathogenesis.

Two PDZ proteins have been identified as targets of the PBM of the E protein of SARS-CoV-1 – PALS1 and Syntenin [2,13]. PALS1 is found in the CRB complex that is involved in maintaining tight junction integrity and cellular polarity [14]. The association between the E PBM and the PALS1 PDZ domain during SARS-CoV-1 infection results in relocalization of PALS1 from tight junctions to the endoplasmic-reticulum-Golgi intermediate compartment. This relocalization disrupts tight junction integrity between lung epithelial cells *in vitro* and this likely contributes to viral pathogenesis and spread of infection between individuals *in vivo*. A recent study examined the binding affinities of PALS1 and peptides representing the PBMs of the E proteins from SARS-CoV-1 and SARS-CoV-2 [15]. The SARS-CoV-2 E PBM peptide has a higher binding affinity to PALS1 than that of the E protein from SARS-CoV-1. However, the significance of this result to viral pathogenesis is uncertain. Syntenin contains tandemly repeated PDZ domains and functions to regulate cytoskeletal–membrane organization, cell adhesion and activation of the p38 MAPK pathway. Syntenin redistributes from the nucleus to the cytoplasm during SARS-CoV-1 infection; thereby, activating p38 MAPK and leading to over expression of inflammatory cytokines [2]. Notably, activation of p38 MAPK and expression of inflammatory cytokines is reduced in infection with SARS-CoV-1 containing a deletion of the E protein PBM. Administration of a p38 MAPK inhibitor increases survival of SARS-1-infected mice, establishing the significance of this pathway and its activation by the E protein via its PBM. Given the conservation of the E protein between SARS-CoV-1 and SARS-CoV-2, an inhibitor of p38 MAPK may have therapeutic benefits for COVID-19 patients. In a recent preprint, the Harty laboratory at the University of Pennsylvania has reported that the PBM of the SARS-CoV-2 E protein targets ZO1 and this may disrupt tight junction integrity; thereby, contributing to pathogenesis.

A common feature of a viral protein with a carboxyl terminal PBM is the ability to target multiple cellular PDZ proteins. Indeed, the influenza A virus NS1 protein PBM targets at least four cellular PDZ proteins: Scribble, Dlg1, MAGI-1 and Lin7C [6–8,16]. PALS1 and Syntenin has been identified as a target of the SARS-CoV-1 E protein PBM, but these proteins are likely to be only two of the many cellular PDZ proteins targeted by the E and Orf3a proteins of SARS-CoV-1 and SARS-CoV-2. Identification of cellular PDZ proteins targeted by these viral PBMs will allow mechanistic studies of viral replication and pathogenesis. Additionally, identification of these PDZ proteins will provide the opportunity for novel therapeutic strategies for infections of SARS-CoV-2 and related coronaviruses that will arise in the future. Finally, the presence of PBMs in the proteins of coronaviruses from animal reservoirs may be a diagnostic for viruses that have potential to be pathogenic in humans.
